# Repair of Mitral Valves with Severe Annular Dilatation and Abundant Leaflet Tissue Using a Prosthetic Ring with a Large Anterior-Posterior Diameter

**DOI:** 10.3390/jcm11061709

**Published:** 2022-03-19

**Authors:** Konstantinos Sideris, Melchior Burri, Joanna Bordne, Keti Vitanova, Bernhard Voss, Markus Krane, Rüdiger Lange

**Affiliations:** 1 Department of Cardiovascular Surgery, German Heart Centre Munich, Technische Universität München, 80636 Munich, Germany; burri@dhm.mhn.de (M.B.); vitanova@dhm.mhn.de (K.V.); voss@dhm.mhn.de (B.V.); lange@dhm.mhn.de (R.L.); 2 Insure (Institute for Translational Cardiac Surgery), Department of Cardiovascular Surgery, German Heart Centre Munich, Technische Universität München, 80636 Munich, Germany; joanna.bordne@gmx.de; 3 Division of Cardiac Surgery, Department of Surgery, Yale University School of Medicine, New Haven, CT 06520, USA; markuskrane@gmx.de; 4 German Heart Center Munich-DZHK Partner Site Munich Heart Alliance, 80636 Munich, Germany

**Keywords:** mitral valve repair, mitral systolic anterior motion, outcomes

## Abstract

Backround: Mitral valve (MV) repair in the case of a large anterior-posterior diameter and redundant valve tissue remains challenging and favors repair with a ring that exhibits a large anterior-posterior diameter. Compared to other available rings, the Medtronic Simulus annuloplasty ring shows the largest anterior-posterior diameter. This study reports for the first time mid-term results using this annuloplasty ring. Methods: Between 11/2015 and 12/2019, a total of 378 patients underwent MV repair for degenerative mitral regurgitation using the Medtronic Simulus ring, according to the following selection criteria: large MV annuli, abundant leaflet tissue (i.e., Barlow disease), and risk for SAM. Results: Overall survival after 5 years was 90.8 ± 4.6%. Five patients required valve-related reoperations because of ring dehiscence (*n* = 1), progression of native valve disease (*n* = 2), dehiscence of quadrangular resection suture (*n* = 1), and endocarditis (*n* = 1). The cumulative incidence of valve-related reoperation at 5 years was 1.3 ± 0.5%. At latest follow-up, echocardiography demonstrated excellent valve function with no/mild MR in 299 patients (94.6%). Two patients (0.6%) had more than moderate MR. No patient developed SAM after repair. Conclusion: Repair of MV with large annuli and abundant leaflet tissue with the Medtronic Simulus annuloplasty ring shows excellent mid-term results regarding reoperation rates and recurrent MR.

## 1. Introduction

**Figure 1:**
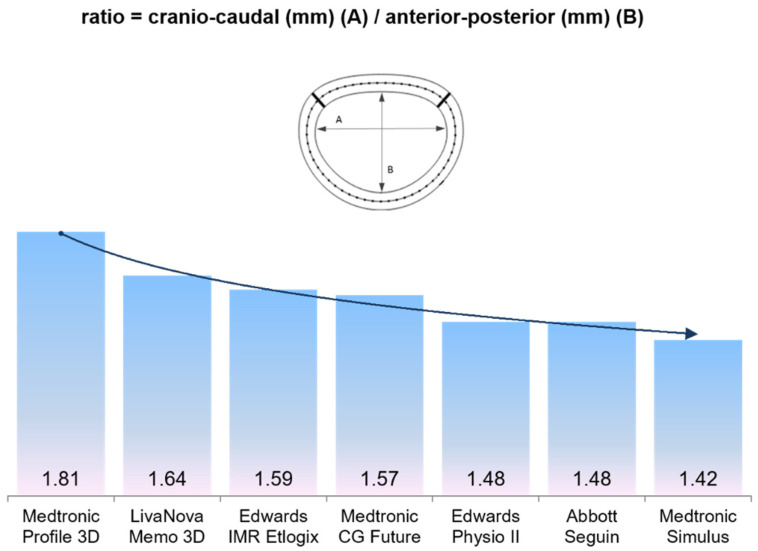
The prosthetic rings available differ substantially in their ratios calculated between the cranial-caudal (A) and anterior-posterior diameter (B). The black arrow demonstrates the reduction of the A/B ratio in the different annuloplasty rings. As an example, a 38 mm size ring was evaluated, which is available from all manufacturers. Dimensions of the rings were provided by the manufacturers Medtronic, LivaNova, and Abbott. The dimensions of the Physio II ring are own measurements because of the restrictive policy of the manufacturer on providing the specifications of the product. LivaNova notes that because of the Nitinol cell core, the Memo3D ring shows a progressive degree of flexibility along the ring from the anterior to the posterior part.

Ring annuloplasty is a fundamental part of mitral valve repair [1]. Guided by the understanding of the function of the mitral valve and the pathophysiological mechanism of mitral regurgitation, a wide variety of annuloplasty devices has been developed so that a specific ring may be chosen for a specific pathology at the discretion of the surgeon. This tailored treatment should address, in particular, the potential risk for the development of postoperative systolic anterior motion (SAM). In the limited data available, the incidence of this complication reaches from 2.1% to 13% [2,3,4,5,6,7]. This risk is increased significantly by factors such as a small left ventricle, a tall posterior leaflet, a narrow aorto-mitral angle, and an enlarged basal septum [7]. Especially for valves with large anterior-posterior diameter and abundant leaflet tissue, predisposed to develop SAM, the application of a suitable annuloplasty ring is important [8]. If the anterior-posterior diameter of the prosthetic ring is too small, the coaptation shifts toward the anterior portion, resulting in partial displacement of the excessive tissue of the anterior leaflet into the LVOT [9]. Particularly in Barlow disease, the anterior leaflet may be too large and exceed the available ring sizes to accommodate the anterior leaflet surface area and the otherwise excessive leaflet tissue. Compared with the reference ratio of a normal mitral valve (CC/AP = 4/3 = 1.33), mitral valves with large anterior-posterior diameter exhibit a ratio of less than 1.33. The smaller this ratio, the larger the anterior-posterior diameter. This elementary knowledge is essential for the choice of the appropriate annuloplasty ring. The available prosthetic rings differ substantially in their ratio calculated between the cranial-caudal and anterior-posterior diameter (Figure 1). Compared to other prosthetic rings, the Medtronic Simulus annuloplasty ring exhibits the largest anterior-posterior diameter, which may be a favorable option for repair of large mitral valves with abundant leaflet tissue and thus avoid SAM.

This is the first study that reports on mid-term results using this specific annuloplasty ring in MV repair. The aim of the study was to evaluate the impact of the Medtronic Simulus annuloplasty ring on repair success and repair durability.

## 2. Materials and Methods

### 2.1. Study Design

All consecutive patients undergoing MV repair and annuloplasty with the Medtronic Simulus annuloplasty ring for degenerative MR at our institution were included in the present study. All relevant patient data were collected in an own database and evaluated retrospectively. Indications for MV repair with the Simulus ring were (1) large mitral valve annuli, (2) abundant leaflet tissue (i.e., Barlow disease), and (3) risk for SAM. The SAM criteria that we followed as described in the literature were end-diastolic diameter < 45 mm, aorto-mitral angle < 120°, coaptation-septum distance < 25 mm, posterior leaflet high > 15 mm, and basal septal diameter ≥ 15 mm [7]. In detail, the cohort consisted of patients who had an oversized valve with a morphological ratio of almost 1:1 (cranio-caudal (CC)/anterior-posterior (AP)). The decision to use a Simulus annuloplasty ring for mitral valve repair was based on the above criteria and according to the preoperative findings of transesophageal echocardiography performed by the cardio anesthesiologist in the operating room.

### 2.2. Study Objectives

The aim of the study was to evaluate the outcome of mitral valve repair with the Simulus annuloplasty ring. Primary endpoints were freedom from recurrent MR and reoperation. Secondary endpoints were survival and functional outcome (defined as NYHA class and rhythm alterations).

### 2.3. Data Acquisition and Echocardiographic Examinations

Baseline data analyses included age, sex, ejection fraction, cardiac rhythm, NYHA class, regurgitation grade, and characteristics of mitral valve pathology. Procedure-related obtained data were recorded in a dedicated database. Echocardiographic examinations at certain time points were required to perform the study. Transthoracic echocardiographic examination was performed as a standard procedure preoperatively and at discharge. Standardized transoesophageal echocardiography was done in all patients during induction and intraoperatively after going off-pump. Follow-up echocardiography was either performed by our outpatient department or by the referring cardiologist. All examinations were performed according to standard procedures recommended by current guidelines [10]. MR was graded as none/trivial (0+), mild (1+), moderate (2+), or severe (3+). A patient’s clinical and hemodynamic status and functional outcome at follow-up were collected from medical records, physical examination at our institution, mailed questionnaires, telephone interviews with the patient, and reports from the referring physicians.

### 2.4. Device Specifications

The Simulus semirigid annuloplasty ring model 800SR consists of a MP35N wire stiffener in the posterior segment running from trigone to trigone. The ring stiffener is enclosed within a close-coiled MP35N spring that passes around the circumference of the annuloplasty ring. The spring is covered by a thin silicone sheath. Braided polyester fabric is used to cover and form the body of the ring. The ring has two green markers to indicate the anterior and posterior trigones. A green demarcation suture runs around the upper face of the ring. The individual ring size (24 to 40 mm in 2 mm increments) refers to the inner circumference between the green trigone markers on the ring. The internal spring and stiffener provide radiographic visualization around the circumference of the ring.

### 2.5. Ethics

The study was conducted in accordance with the Declaration of Helsinki and was approved by the local governmental ethics committee (approval reference number: 564/16 S, 14 December 2016). Written informed consent was obtained from each participant.

### 2.6. Statistical Analysis

Statistical analysis was performed with IBM SPSS 22 (SPSS Inc., Chicago, IL, USA) and R (version 3.5.2; R Foundation for Statistical Computing, Vienna, Austria). Normally, distributed continuous variables are presented as mean ± standard deviation (SD). Categorical variables are presented as number (%). Overall survival was analyzed with Kaplan-Meier methods and the log-rank test. Freedom from reoperation was analyzed with cumulative incidence functions for competing risks.

## 3. Results

### 3.1. Patient Characteristics

**Figure 2:**
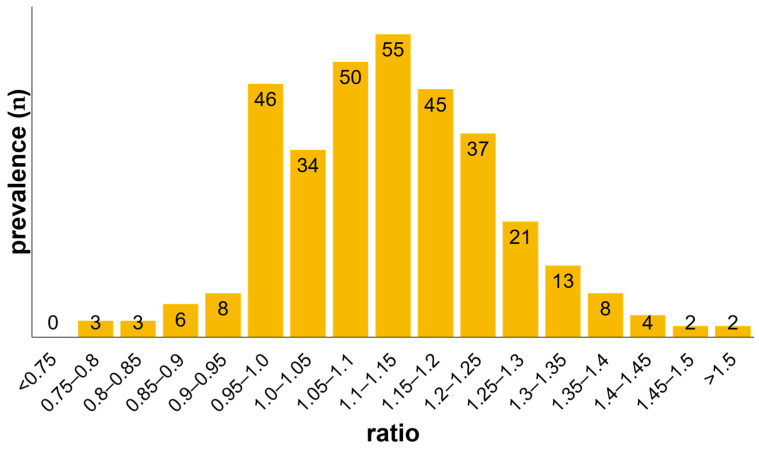
Mitral valve morphology at baseline (ratio = C-C/A-P).

Between 11/2014 and 12/2019, a total of 1023 mitral valve repair procedures were performed at our institution. During this period, 378 (37%) patients with degenerative MR were treated using the Simulus semirigid annuloplasty ring. Preoperative valve pathology showed posterior leaflet prolapse in 244 patients (64.6%), anterior leaflet prolapse in 19 (5%), and bileaflet prolapse in 111 (29.4%). In addition, 121 patients (32%) presented with Barlow’s disease. The baseline data are summarized in Table 1. In detail, the cohort consisted of patients who either fulfilled one or more SAM criteria or had an oversized valve with a morphological ratio of almost 1:1 (cranio-caudal (CC)/anterior-posterior (AP)). Figure 2 shows the distribution of the cranio-caudal to anterior-posterior ratios of MV at baseline. Compared with the reference ratio of a normal mitral valve (CC/AP = 4/3 = 1.33), 95% of our patients had a ratio of less than 1.33. The assessment of MV dimensions showed that the mean C-C diameter was 42.9 mm (range, 28–63 mm) and the mean A-P diameter was 38.4 mm (range 27–63 mm), which corresponds to a ratio of 1:1.

### 3.2. Operative Procedure

In 175 patients (46.3%) the approach was a median sternotomy and in 203 (53.7%) a right anterolateral thoracotomy. All procedures were performed on cardiopulmonary bypass protecting the heart by application of cold crystalloid cardioplegia under moderate systemic hypothermia. The mitral valve was exposed via a left atrial or a trans-septal approach. The results of the detailed valve analysis are displayed in Table 2.

### 3.3. Follow-Up

**Figure 3:**
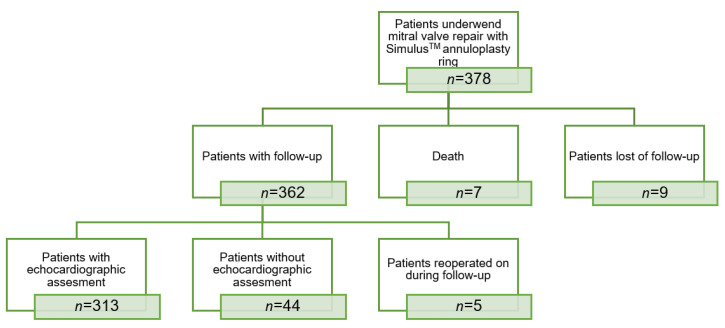
Study population at latest follow-up.

Follow-up was complete in 97.6% of patients with a mean follow-up of 2.4 ± 1.4 years. Nine patients were lost to follow-up. Study endpoints were analyzed according to the “Guidelines for Reporting Morbidity and Mortality after Cardiac Valvular Operations” [11]. Figure 3 summarizes the status of the study population at latest follow-up.

### 3.4. Survival

**Figure 4:**
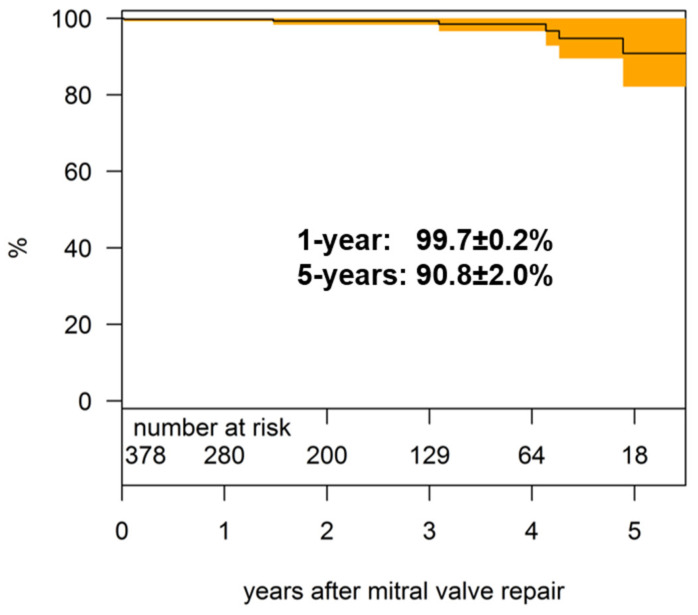
Survival.

We observed no intraoperative deaths. The 30-day mortality was 0.3% (1/378). At last follow-up, 362 patients (95.8%) were alive with an overall survival of 99.7 ± 0.2% and 90.8 ± 4.6% after 1 and 5 years, respectively (Figure 4). Seven patients (1.9%) died during follow-up (mean time 3.4 ± 2.0 years). The cause of death could be determined in four patients (two patients with cardiac-related and two others with noncardiac-related causes).

### 3.5. Echocardiographic Results

**Figure 5:**
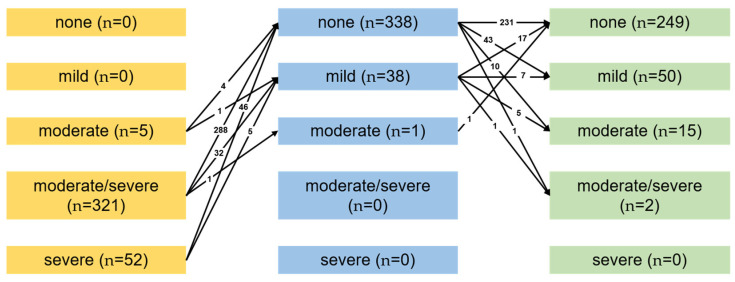
Comparison of MR preoperatively, at hospital discharge, and at latest follow-up. The black arrows show the qualitative and quantitative (—n—) change in mitral regurgitation. We included only patients with complete echocardiographic follow-up. Patients who died in-hospital or during the follow-up period, patients who were lost to follow-up, and patients who were reoperated on have been excluded. MR, mitral regurgitation.

Our results show that at discharge 338 patients (89.7%) had no residual MR, 38 (10.1%) remained with mild MR, and 1 (0.3%) with moderate MR, whereas no patient had more than moderate MR. At latest follow-up (2.3 ± 1.5 years), echocardiographic examinations of 316 patients (87.6%) were available. The results show no MR in 249 patients (78.8%), mild in 50 (1.6%), and moderate in 15 (4.7%). Two patients (0.6%) presented with more than moderate mitral regurgitation (Figure 5). No patient had a significant stenosis.

### 3.6. Reoperations

**Figure 6:**
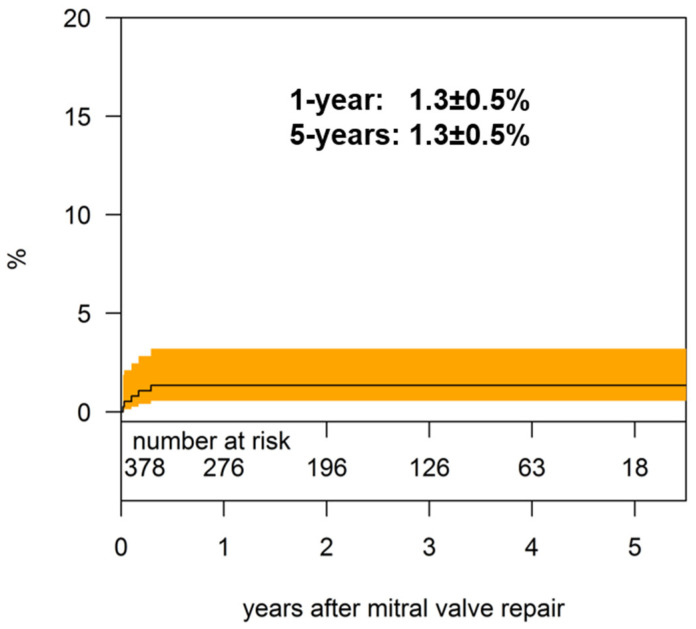
Cumulative incidence of reoperation.

The data of the reoperated patients are summarized in Table 3. The cumulative risk for mitral valve-related reoperations was 1.3 ± 0.5% and 1.3 ± 0.5% at 1 and 5 years, respectively (Figure 6).

### 3.7. Functional Status

**Figure 7:**
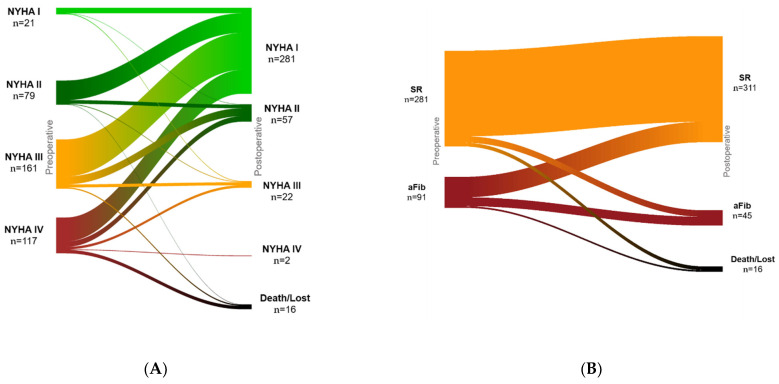
Patients functional status preoperative and at discharge. (**A**): NYHA (New York Heart Association) class, (**B**): cardiac rhythm. SR: sinus rhythm, aFib: atrial fibrillation.

Of the patients, 73.5% presented prior to the operation with NYHA class III or IV. Figure 7A shows the changes in NYHA class at latest follow-up among patients with data available at both time points. The percentage of patients in sinus rhythm increased from 75.3% at baseline to 83.67% at latest follow-up. The incidence of atrial fibrillation decreased from 24.5% at baseline to 12.1% following mitral valve repair with the Simulus annuloplasty ring. The changes in rhythm during follow-up are illustrated in Figure 7B. The number of patients in whom a MAZE procedure was performed was 67 (17.7%).

## 4. Discussion

Compared to other annuloplasty devices, the Simulus ring exhibits the smallest cranio-caudal to anterior-posterior ratio, and thus possesses ideal properties for mitral valve repair in cases of large mitral valve annuli and abundant leaflet tissue (Figure 1). The present study shows a low incidence of MV-related reoperation and recurrent MR. An additional important finding was the absence of postprocedural SAM, which may be attributed to the special geometry of the Simulus ring.

### 4.1. Systolic Anterior Motion after Mitral Valve Repair

Systolic anterior motion was first recognized as a serious complication after mitral valve reconstruction in the late 1970s [12]. Since this first description, various publications followed but estimates of the incidence and recommendations for the management of SAM vary. In a cohort of 2076 patients, Brown et al. found SAM in 8.4% of the patients [3]. Ashikhmina observed SAM immediately after cardiopulmonary bypass in 13% of the patients (98 of 761 patients) [2]. Loulmet and colleagues reported an incidence of SAM of 4% (77 of 1906) [4] and Noack et al. of 2.1% (10/486) [6]. Some morphologic criteria of valves may be predisposed to develop SAM after repair, such as abundant leaflet tissue, steep aortic-mitral angle, septal hypertrophy, and small c-sept distance. A procedural risk factor for the development of postoperative SAM is an inadequate reduction of the height of the posterior leaflet. Adams et al. published a series of 67 patients who received mitral valve repair for Barlow disease [9]. Using large annuloplasty rings and predominant resection of the posterior leaflet only 1 out of 67 patients developed postoperative SAM. However, the authors admitted that the ring used in this study had reached its limit in some cases, in which the anterior leaflet was so large that sizing on the basis of the area of the anterior leaflet became inadequate.

The Simulus annuloplasty ring used in our study proved to be particularly advantageous because the anterior-posterior diameter of the Simulus ring is larger than in other semi-rigid rings (Figure 1). In the present study, the patients either fulfilled one or more SAM criteria or exhibited a large annulus with a morphological ratio of almost 1:1 (cranio-caudal (CC)/anterior-posterior (AP)). Of our patients, 95% showed a ratio of less than 1.33 (Figure 2). Despite these risk factors, we did not observe any SAM after repair with the Simuls ring and attribute this to its specific geometry (Figure 1).

### 4.2. Hemodynamic, Survival and Redo

In our cohort, we were able to demonstrate a very low rate of reoperation and recurrent MR. With the Simulus annuloplasty device, the 30-day mortality in our series was 0.3%, the survival rate at 5 years was 90.8%, and the incidence of reoperation at 5 years was 1.3%. This is in accordance with the current literature regarding MV repair in degenerative MR. David et al. reported a reoperation rate of 2.7% at 5 years and a freedom from more than moderate MR of 96.3% at 5 years [13]. Chan and colleagues published a study with 97 patients who underwent MV repair using the Physio annuloplasty ring [14]. They observed a 30-day mortality of 2.1% and reported a survival and freedom from reoperation at 4 years of 93.6 and 93.8%, respectively. Recently, Noack et al. reported mid-term results of mitral valve repair using the Physio II ring [6]. They demonstrated a freedom from MV related reoperations of 96.3 and 94% at 1 and 4 years, respectively.

### 4.3. Implications for the Use of the Simulus Ring

The present investigation demonstrates that mitral valve repair using the Simulus ring leads to durable functional results with very low recurrent MR rates in a patient cohort with large mitral annuli (Figure 2). Furthermore, we did not observe SAM, although in most cases no resection of the mitral valve leaflets was performed. This point is essential because large valves with abundant tissue are at higher risk to develop SAM. In our opinion, these results are most likely achieved because of the unique geometric characteristics of the ring. Compared to other devices, the Medtronic Simulus annuloplasty ring has the largest anterior-posterior diameter and may thus be the ideal device for the repair of mitral valves with large mitral valve annuli and abundant leaflet tissue. Certainly, the absence of SAM cannot be attributed only to the unique geometrical characteristics, since we used other techniques known to avoid SAM. Nevertheless, in our opinion the geometrics of the Simulus annuloplasty ring is the crucial reason. This result needs to be validated with prospective randomized trials.

## 5. Conclusions

In a specific subset of patients, MV repair with the Simulus annuloplasty ring shows excellent mid-term results regarding reoperation rates and recurrent MR. For this reason, we consider the Simulus ring an important adjunct to the armamentarium of annuloplasty devices for a special indication.

## 6. Limitations

The present study has several limitations. First, this is a single-center, nonrandomized retrospective study. Second, the ring was used in specific pathologies, which led to a degree of patient selection. Furthermore, various techniques were used for MV repair. Finally, echocardiographic follow-up was performed by the local cardiologists. This may lead to different interpretations of the results. In addition, the measurements are partly incomplete. Therefore, it was difficult to evaluate the effect of the ring on LV remodeling.

## Figures and Tables

**Table 1:** Baseline data.

Baseline Data	
Patients (*n*)	378
Age, ^a^ years (mean ± SD)	58 ± 11.8
Male, *n* (%)	273 (72.2)
Ejection fraction, %	61 ± 7
Atrial fibrillation, *n* (%)	91 (24.0)
NYHA class, *n* (%)	
I	21 (5.5)
II	79 (20.9)
III	161 (42.6)
IV	117 (30.9)
Pulmonary hypertension, *n* (%)	68 (18.0)

^a^ Results are presented as mean ± standard deviation, MV: mitral valve, NYHA: New York Heart Association.

**Table 2:** Procedural data.

Procedural Data	
Mechanism of MR, *n* (%)	
Flail/prolapse PML	244 (64.4)
Flail/prolapse AML	19 (5.0)
Bileaflet prolapse	111 (29.4)
Barlow’s disease	121 (32.0)
dilatation only	4 (1.0)
Chordal replacement (ePTFE sutures)	266 (70.4)
CR with ePTFE sutures + closure of CLI	91 (24.1)
Closure of CLI	123 (32.5)
PML resection	104 (27.5)
AML resection	5 (1.3)
Alfieri stich	57 (15.1)
Minimally invasive, *n* (%)	203 (53.7)
Aortic cross-clamp time, min ^a^	94.1 ± 31.2
Ring size	
28	8 (2.1)
30	22 (5.8)
32	60 (15.9)
34	86 (22.8)
36	64 (16.9)
38	69 (18.3)
40	69 (18.3)
Concomitant procedures, *n* (%)	175 (46.3)
ASD/PFO closure	69 (18.3)
CABG	33 (8.7)
Tricuspid valve repair/replacement	86 (22.7)
Aortic valve repair/replacement	20 (5.3)
Ablation of Afib	67 (17.7)
Congenital	3 (0.8)
Aortic surgery	6 (1.5)

CR: chordal replacement, ePTFE: expanded polytetrafluoroethylene, PML: posterior mitral valve leaflet, AML: anterior mitral valve leaflet, CLI: cleft-like indentation, ^a^ results are presented as mean ± standard deviation. ASD: atrial septum defect, PFO: persistent foramen ovale, CABG: coronary artery bypass graft, Afib: atrial fibrillation.

**Table 3:** Data of reoperated patients.

Patient	Age at Operation (Years)	Procedure at MV	Concomitant Procedure	Time to Redo (Days)	Cause of Redo	Procedure Performed
1	66	Simulus 36, triangular resection	Aortic Valve replacement	37	Ring dehiscence	Ring refixation
2	43	Simulus 40, CR PML, closure of CLI PML	none	62	Progression of native valve disease	Re-repair (triangular Resektion AML, CR AML
3	47	Simulus 38, Alfieri stich	none	11	Progression of native valve disease	MV replacement
4	49	Simulus 36, CR PML	none	106	Endocarditis	MV replacement
5	58	Simulus 34, quadrangular resection PML	PFO closure	7	Dehiscence of quadrangular resection suture	MV replacement

MV: mitral valve, CR: chordal replacement, PML: posterior mitral valve leaflet, AML: anterior mitral valve leaflet, CLI: cleft-like indentation, PFO: persistent foramen ovale.

## Data Availability

The data presented in this study are available on reasonable request from the corresponding author.

## References

[1] Oliveira J.M., Antunes M.J. (2006). Mitral valve repair: Better than replacement. Heart.

[2] Ashikhmina E., Schaff H.V., Daly R.C., Stulak J.M., Greason K.L., Michelena H.I., Fatima B., Lahr B.D., Dearani J.A. (2021). Risk factors and progression of systolic anterior motion after mitral valve repair. J. Thorac. Cardiovasc. Surg..

[3] Brown M.L., Abel M.D., Click R.L., Morford R.G., Dearani J.A., Sundt T.M., Orszulak T.A., Schaff H.V. (2007). Systolic anterior motion after mitral valve repair: Is surgical intervention necessary?. J. Thorac. Cardiovasc. Surg..

[4] Loulmet D.F., Yaffee D.W., Ursomanno P.A., Rabinovich A.E., Applebaum R.M., Galloway A.C., Grossi E.A. (2014). Systolic anterior motion of the mitral valve: A 30-year perspective. J. Thorac. Cardiovasc. Surg..

[5] Manabe S., Kasegawa H., Arai H., Takanashi S. (2018). Management of systolic anterior motion of the mitral valve: A mechanism-based approach. Gen. Thorac. Cardiovasc. Surg..

[6] Noack T., Sieg F., Cuartas M.M., Spampinato R., Holzhey D., Seeburger J., Borger M.A. (2021). Clinical outcomes after mitral valve repair with the physio ii annuloplasty ring. Thorac. Cardiovasc. Surg..

[7] Varghese R., Itagaki S., Anyanwu A.C., Trigo P., Fischer G., Adams D.H. (2014). Predicting systolic anterior motion after mitral valve reconstruction: Using intraoperative transoesophageal echocardiography to identify those at greatest risk. Eur. J. Cardiothorac. Surg..

[8] Alfieri O., Lapenna E. (2015). Systolic anterior motion after mitral valve repair: Where do we stand in 2015?. Eur. J. Cardiothorac Surg.

[9] Adams D.H., Anyanwu A.C., Rahmanian P.B., Abascal V., Salzberg S.P., Filsoufi F. (2006). Large annuloplasty rings facilitate mitral valve repair in barlow’s disease. Ann. Thorac. Surg..

[10] Lancellotti P., Tribouilloy C., Hagendorff A., Popescu B.A., Edvardsen T., Pierard L.A., Badano L., Zamorano J.L., Scientific Document Committee of the European Association of Cardiovascular Imaging (2013). Recommendations for the echocardiographic assessment of native valvular regurgitation: An executive summary from the european association of cardiovascular imaging. Eur. Heart J. Cardiovasc. Imaging.

[11] Akins C.W., Miller D.C., Turina M.I., Kouchoukos N.T., Blackstone E.H., Grunkemeier G.L., Takkenberg J.J., David T.E., Butchart E.G., Adams D.H. (2008). Guidelines for reporting mortality and morbidity after cardiac valve interventions. Eur. J. Cardiothorac. Surg..

[12] Termini B.A., Jackson P.A., Williams C.D. (1977). Systolic anterior motion of the mitral valve following annuloplasty. Vasc. Surg..

[13] David T.E., Armstrong S., McCrindle B.W., Manlhiot C. (2013). Late outcomes of mitral valve repair for mitral regurgitation due to degenerative disease. Circulation.

[14] Chan D.T., Chiu C.S., Cheng L.C., Au T.W. (2006). Mitral valve annuloplasty with carpentier-edwards physio ring: Mid-term results. Asian Cardiovasc. Thorac. Ann..

